# A novel approach for low-temperature synthesis of nanostructured rutile-like Ti_1−*x*_Fe_*x*_O_2_ solid solutions

**DOI:** 10.1039/d4ra03274h

**Published:** 2024-09-04

**Authors:** Franklin J. Méndez, Alejandro Herrera-González, Antonio Morales, Xim Bokhimi

**Affiliations:** a Instituto de Física, Universidad Nacional Autónoma de México, Ciudad Universitaria Coyoacán Ciudad de México 04510 Mexico bokhimi@fisica.unam.mx

## Abstract

We present a straightforward method for synthesizing rutile-like Ti_1−*x*_Fe_*x*_O_2_ solid solutions at 90 °C, with *x* = 0.02, 0.04, 0.06, and 0.08. Additionally, for reference, we synthesized Fe-free rutile under identical conditions. All samples were characterized using XRPD, Rietveld refinement, elemental analysis, and specific surface area. Further characterization of the pure rutile and the solid solution with *x* = 0.04 was conducted using HRTEM, SEM-EDS, Raman spectroscopy, UV-vis DRS, and Mössbauer spectroscopy. The results indicated that Fe atoms incorporated into the crystalline structure of rutile, replacing Ti atoms. All phases exhibited a tetragonal crystalline structure with lattice parameters that increased with Fe content. Rietveld refinement and the electron microscopy revealed that the crystallites had a morphology elongated along the *c*-axis. Experimental evidence showed that the incorporation of iron into the crystalline structure altered the optical properties, as corroborated through DFT calculations on a Fe-free rutile cluster and one doped with Fe. These calculations also suggest enhancement of the stability of the solid solutions.

## Abstract 2

Rutile-like Ti_1−*x*_Fe_*x*_O_2_ solid solutions were synthesized at 90 °C with crystallites elongated along the *c*-axis.

## Graphical abstract

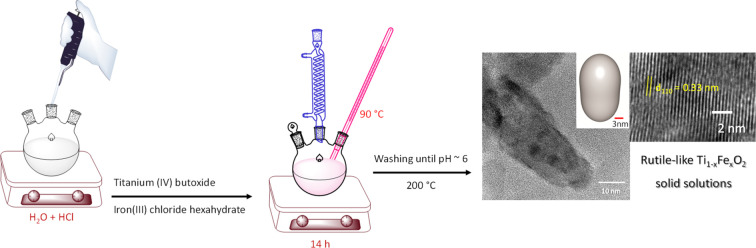

## 1. Introduction

Anatase, rutile, and brookite are the three well-known TiO_2_ polymorphs, each distinguished by its atom arrangement, and particularly the presence of distorted TiO_6_ octahedra chains.^1^ Anatase is favored for its thermodynamic stability at smaller particle sizes, while rutile is the most stable phase at larger particles, and brookite is predominant in the intermediate size range.^2^ The polymorph type significatively influences the properties of titania, leading to extensive research into their phase transformations.^3^ Typically, anatase is the initial crystalline phase, with rutile irreversibly forming through calcination at around 600 °C,^3^ although its formation has been reported across a broader range of 400 to 1200 °C.^4–6^ Transitions from brookite to rutile are less frequently studied, but Li and Ishigaki have reported this transformation occurring between 500 and 600 °C.^7^ Thus, it is evident that the synthesis temperature plays a crucial role in determining the TiO_2_ polymorph formed.

Rutile has received less attention due to its lower photoelectrochemical activity.^8^ However, it offers several advantages such as excellent chemical resistance, light-scattering properties, and a higher refractive index,^9^ making it valuable, particularly when doped with cations like Fe, which exhibit characteristic colors depending on their oxidation state,^10^ to form solid solutions. Despite these benefits, studies on Fe-modified rutile solid solutions are limited. Tena *et al.*^11,12^ synthesized rutile-like Fe_*x*_Ti_1−2*x*_M_*x*_O_2_ (M = Nb, Ta) and (M,V)-TiO_2_ (M = Al, Cr, Fe) solid solutions, noting that the gel method allows for synthesis at lower temperatures compared to the ceramic method. The electrical conductivity of these solid solutions was found to be influenced by the presence of Fe^2+^ ions and local distortions in M−O bond lengths.^13^ Filipek and Dabrowska expanded this work by synthesizing a new solid solution, Fe_1−*x*_Cr_*x*_VSbO_6_, with a rutile-type structure.^14^ Brink *et al.* further explored nonstoichiometric rutile-type solid solutions in the Fe^II^F_2_-Fe^III^OF system, identifying distinct solid solution regions and providing explanations for observed diffraction phenomena.^15^ However, these rutile-type solid solutions were typically synthesized at high temperatures, up to 1000 °C. Therefore, our study focuses on developing a novel method to obtain a series of rutile-like Ti_1−*x*_Fe_*x*_O_2_ solid solutions at comparatively lower temperatures.

## 2. Experimental

Rutile-like Ti_1−*x*_Fe_*x*_O_2_ solid solutions, with *x* = 0.02, 0.04, 0.06, and 0.08, were synthesized at low temperature. In a typical experiment, a mixture of 23 mL of deionized water (H_2_O, Hycel) and 11.32 mL of hydrochloric acid (HCl, 36.5–38.0%, Baker Analyzed® ACS, J.T. Baker®) was prepared in a three-necked flask reactor at room temperature. HCl plays a crucial role in facilitating the hydrolysis of the Ti precursors, thereby promoting the formation of the rutile crystal structure at low temperatures.^16,17^ Titanium(iv) butoxide (Ti[O(CH_2_)_3_CH_3_]_4_, reagent grade 97%, Sigma-Aldrich) was then added dropwise, followed by the addition of iron(iii) chloride hexahydrate (FeCl_3_·6H_2_O, ACS reagent, 98.0–102% RT, Sigma-Aldrich), with each addition being homogenized for 30 minutes. The amount of Fe precursor was adjusted to achieve the desired concentration. The resulting solution was heated to 90 °C and maintained at this temperature for 14 h. During this time, a yellow precipitate formed, which was subsequently recovered by centrifugation. To remove Cl^−^ ions and obtain the final product, the solid was washed with deionized water and centrifuged at 10 000 rpm until the pH of the dispersion reached approximately 7. The solid was then dried at 90 °C. As a reference sample, Fe-free rutile was prepared under the same synthesis conditions, resulting in a white powder.

## 3. Results and discussion

**Fig. 1:**
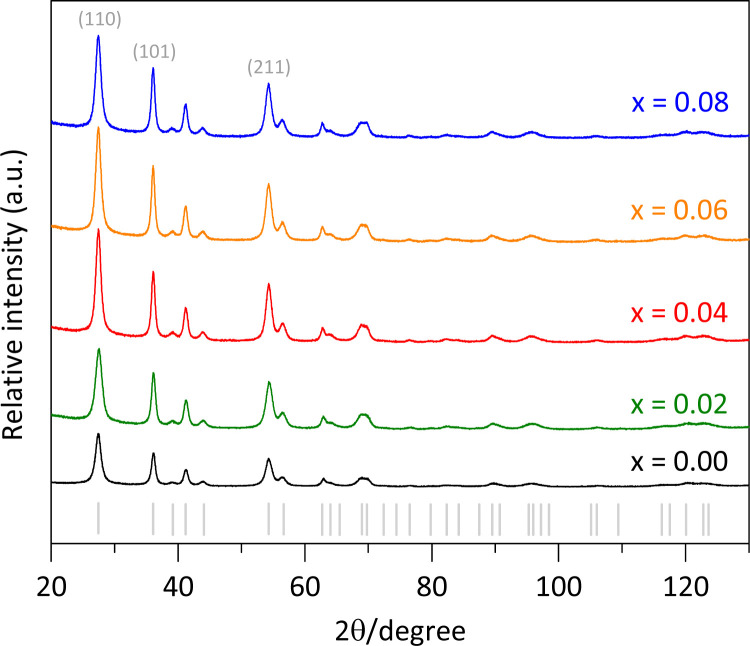
X-ray powder diffraction patterns of pure rutile and the Ti_1−*x*_Fe_*x*_O_2_ solid solutions.

The synthesized pure rutile and the solid solutions were characterized using X-rays powder diffraction (XRPD) with a BRUKER D8 Advance diffractometer, operating in Bragg–Brentano geometry (*θ*–*θ* configuration), with Cu Kα radiation, a Ni filter, and a detector equipped with 192 silica strips (BRUKER, LynxEye). Diffraction intensities were measured as a function of the diffraction angle from 20° to 130°, with a 2*θ* step of 0.0194° and a measurement time of 211 seconds per point. The resulted diffractograms are shown in Fig. 1, where all the diffraction peaks correspond to a rutile-like phase (ICDD file 01-071-0650).^18^ No other Fe-based phases or TiO_2_ polymorphs were detected.

**Fig. 2:**
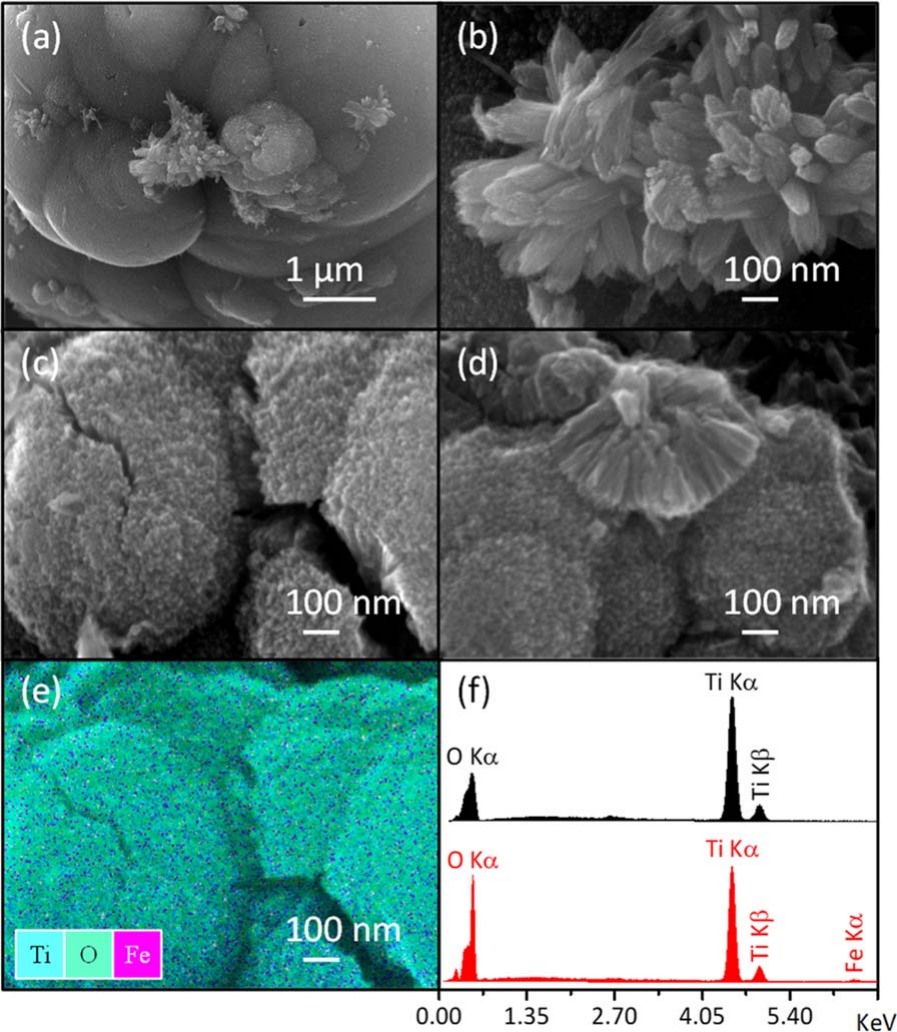
SEM images of pure rutile (a and b) and the solid solution with *x* = 0.04 (c and d) obtained using an OXFORD-ISIS model 7585 microanalyzer coupled with a JEOL JSM-7800F SEM. Total elemental mapping of the Fe-modified sample (e) and EDS spectra (f) are also included.

In addition to the sample color, the presence of Fe was confirmed through SEM-EDS analysis (Table 1/Fig. 2f) and elemental mapping (Fig. 2e). Due to the low Fe loading in the synthesis medium, some uncertainty was introduced in the elemental analysis (Table 1), leading to a slightly higher measured Fe content than the nominal values. However, the high dispersion of Fe was evident, as the elemental mapping showed a uniform distribution throughout the sample with no visible aggregation (Fig. 2e). This fact supports the formation of the Fe-doped rutile-type structure at low temperatures.

**Table 1:** EDS elemental analysis, specific surface area, average microstrain (*ε*), and average crystallite size of pure rutile and the Ti_1−*x*_Fe_*x*_O_2_ solid solutions

Ti_1−*x*_Fe_*x*_O_2_ solid solution	Fe loading (wt%)		*S*_BET_ b (m^2^ g^−1^)	*ε* c (%)	Average crystal size^c^ (nm)
	Nom.	Exp.^a^			
*x* = 0.00			114	1.34(2)	12.3(1)
*x* = 0.02	1.4	2.0(2)	115	1.29(2)	12.0(1)
*x* = 0.04	2.8	3.7(4)	94	1.23(1)	14.9(1)
*x* = 0.06	4.3	6.0(4)	93	1.34(1)	15.0(1)
*x* = 0.08	5.7	7.3(6)	97	1.33(2)	14.2(1)

a Elemental analysis was obtained with an OXFORD-ISIS model 7582 microanalyzer coupled with a JEOL JSM-7800F SEM.

b Specific surface areas were determined from nitrogen physisorption data using the Brunauer–Emmett–Teller multipoint method in an AUTOSORB-1 analyzer from QUANTACHROME®.

c Average microstrains and crystallite size values were determined from Rietveld refinement.

Additionally, a higher intensity for the (110) peak compared to the (211) peak was observed in Fig. 1. The expected (110)/(211) ratio from the referenced ICCD card is approximately 1.66, but the observed ratios were slightly lower, decreasing from 1.31 to 1.11 as the Fe content increased from *x* = 0.00 to 0.08. This suggests predominant particle growth perpendicular to the [110] direction. These findings are consistent with the SEM images (Fig. 2b and d), which reveal that the crystallites are oriented along the *c*-axis.

**Fig. 3:**
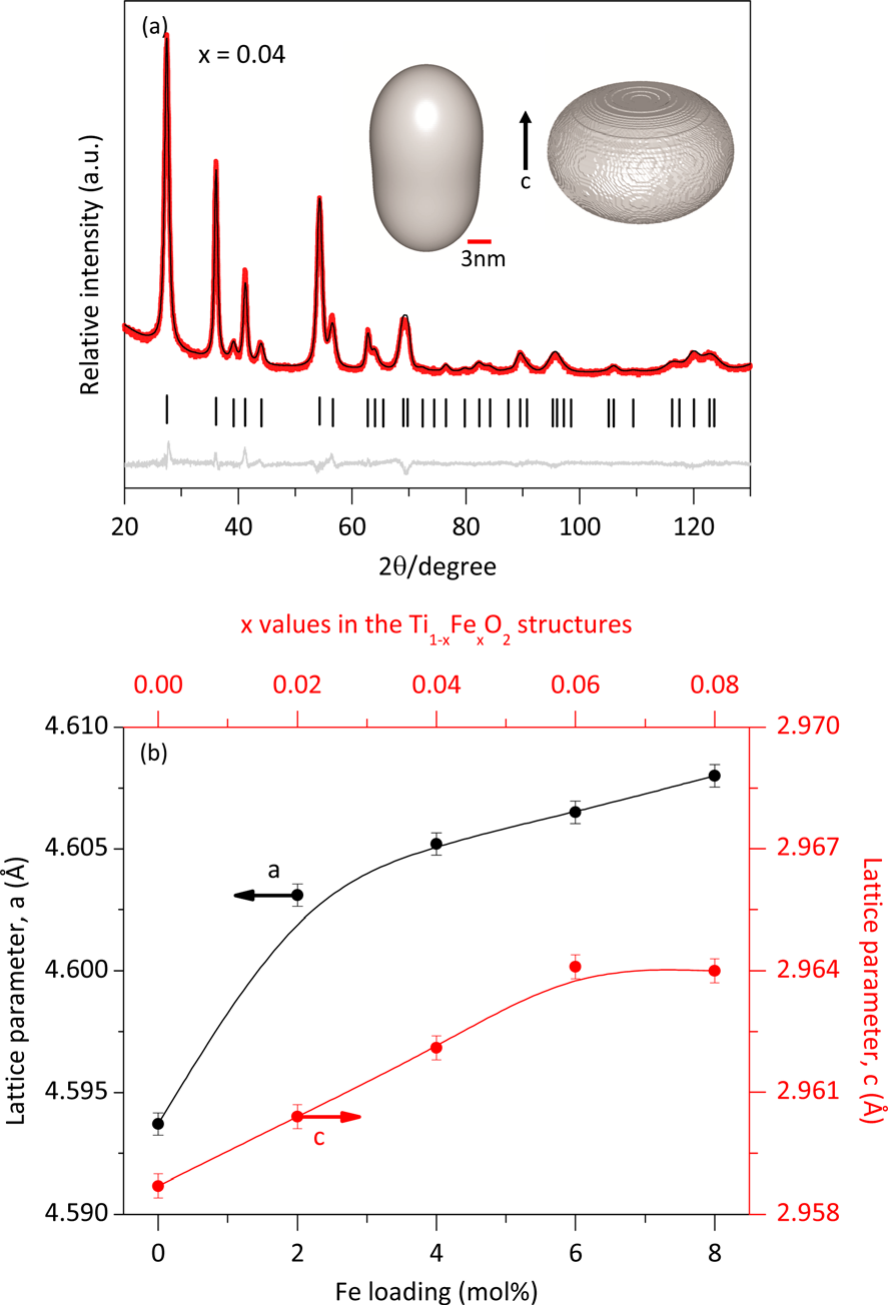
Rietveld refinement of the solid solution with *x* = 0.04 (a); the inset presents the calculated morphology of the crystallite and its microstrain distribution. Relationship between lattice parameters and Fe loading (b).

The crystalline structures were refined using the Rietveld method^19^ implemented in TOPAS software version 7. The background model employed a polynomial function, including constant, linear, quadratic, and cubic terms in 2*θ*, along with additional terms (1/2*θ*) and (1/2*θ*)^2^. As no Fe-containing phases other than the solid solutions were observed in the XRPD analysis, we conclude that all Fe atoms were integrated into the rutile lattice by substituting Ti atoms according to the doping concentration. The experimental diffraction pattern (red lines) and the modelled one (black line) for a sample of the solid solution with *x* = 0.04 are shown in Fig. 3a. The minimal difference (gray lines) between the experimental and modeled patterns provides strong evidence for the formation of a single rutile-like phase.

Furthermore, the refinement confirmed that the crystalline structure of both rutile and the solid solutions was tetragonal with an atomic distribution described by the space group *P*4_2_/*mnm*. For the pure rutile phase, the refined lattice parameters were *a* = *b* = 4.5941 Å, *c* = 2.9589 Å, with *α* = *β* = *ϒ* = 90°, consistent with previous reports.^20^ However, the incorporation of Fe atoms caused slight modifications of the crystalline structure of rutile.

The data obtained from the Rietveld refinement enabled the generation of representative images of the crystallite morphology and microstrain distribution (inset Fig. 3a). The crystallites exhibit an elongated morphology along the *c*-axis. This elongation may be attributed to the crystalline structure of rutile and the solid solutions, characterized by chains of edge-bonded TiO_6_ octahedra aligned along the *c*-axis. The refined lattice parameters as a function of Fe content closely followed Vegard's law.^21^ Meanwhile, Table 1 indicates that the average microstrain (*ε*) remained nearly constant, regardless of Fe concentration. This suggests that the average microstrain values are primarily by the small size of the crystallites, which have numerous distorted unit cells near their surface. The additional deformation of unit cells due to Fe atoms incorporation was minimal. Interestingly, the microstrain distribution (Fig. 3a) was found to be greater in the direction perpendicular to the *c* axis.

**Fig. 4:**
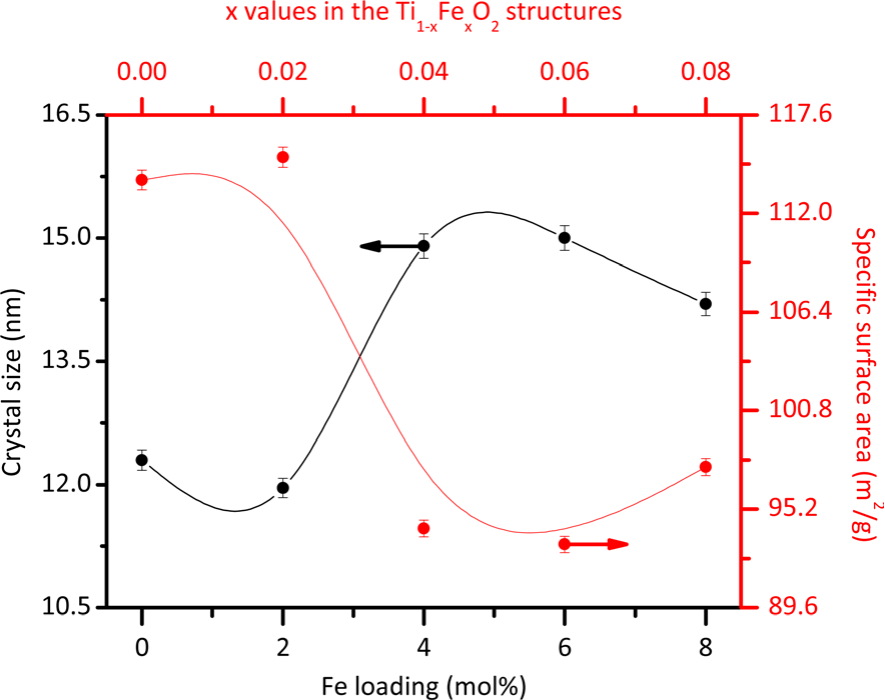
Average crystallite size and specific surface area obtained from the Rietveld refinement for the different Fe loadings.

The average morphology of the crystallites was modelled using a symmetrized harmonics expansion for crystallite size refinement.^22^ The specific surface area was determined using both Rietveld refinement (Fig. 4) and nitrogen physisorption (Table 1). As shown in Fig. 4 an increase in Fe loading leads to an increase in crystallite size and a corresponding decrease in surface area, highlighting a clear inverse relationship between crystal size (black line) and specific surface area (red line). Notably, crystallite size and surface area remain relatively unaffected up to Fe concentrations of 4 mol% Fe (*x* = 0.04). Beyond this concentration, crystallite size increases and surface area decreases more significantly.

Previous studies have also explored these effects. Singh *et al.*^23^ reported that Fe doping tends to increase the size of rutile crystallites. However, Xue *et al.*^24^ demonstrated that at higher doping levels, Fe can inhibit grain growth by increasing the growth activation energy. In line with this, the introduction of Fe ions into the rutile lattice may facilitate the growth of larger crystallites by reducing the number of nucleation sites. This occurs because Fe^3+^ ions, with a larger ionic radius than Ti^4+^ ions, cause lattice distortion, which can reduce the overall nucleation rate and promote the growth of existing crystallites rather than the formation of new ones. Additionally, Fe ions may act as a flux during synthesis, lowering the activation energy for atom migration and enhancing the coalescence of smaller crystallites into larger ones. It is important to note that the specific surface area values obtained through nitrogen physisorption (Table 1) are slightly lower than those obtained from Rietveld refinements (Fig. 4). This discrepancy could be due to the physisorption technique's limited access to all crystallite faces. However, both analyses consistently demonstrate that the specific surface area decreases with increasing Fe content.

**Fig. 5:**
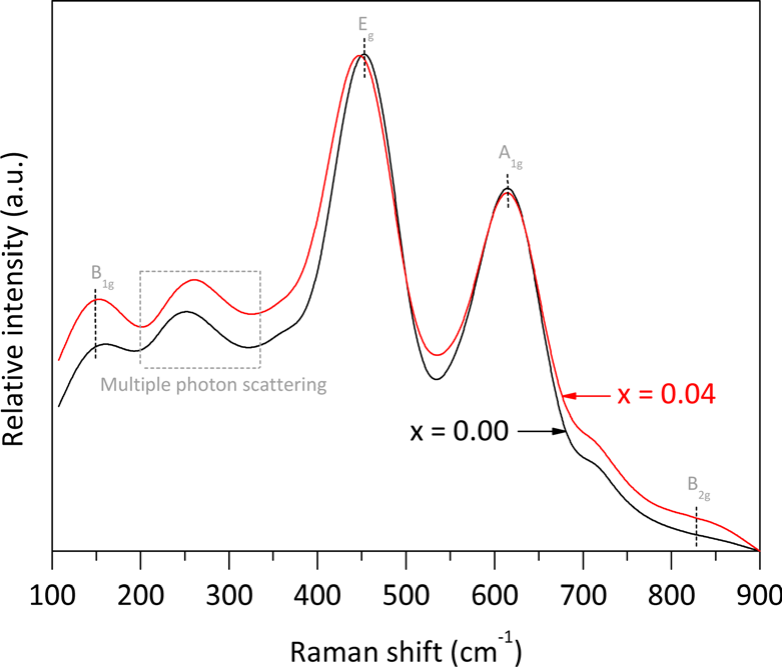
Raman spectra of pure rutile and the solid solution with *x* = 0.04 obtained using an ANTON PAAR spectrometer model Cora 5000 (*λ* = 1064 nm, 300 mW).

The pure rutile and the solid solution with *x* = 0.04 were selected to investigate their vibrational and electronic properties, as well as the Fe valence, Fe local environment, and their microstructures using Raman spectroscopy, UV-vis DRS, Mössbauer spectroscopy, and HRTEM, respectively. According to Porto *et al.*,^25^ rutile phase is expected to exhibit four allowed active vibration modes with symmetries B_1g_, E_g_, A_1g_, and B_2g_, which can be observed using Raman spectroscopy. In our analysis of pure rutile, three peaks were observed at 150.2, 451.6, and 614.9 cm^−1^, corresponding to the B_1g_, E_g_, and A_1g_ symmetries, respectively. These three bands are associated with the symmetric bending, symmetric stretching, and asymmetric bending vibrations of O–Ti–O bonds in the crystalline structure.^26^ It is important to note that the characteristic mode of vibration at approximately 826.0 cm^−1^, associated with the B_2g_ symmetry, was not clearly observed due to its very weak intensity. Additionally, a broad peak between 200 and 330 cm^−1^ was detected, attributed to multiple phonon scattering processes.^27^ For the solid solution with *x* = 0.04, the same Raman signals were observed, but with slight shifts in the main peak positions and some broadening compared to pure rutile. Notably, the E_g_ band shifted from 451.6 to 448.2 cm^−1^, resulting in a Δ*ν* value of approximately −3.4 cm^−1^. According to Loan *et al.*,^28^ when Fe^3+^ ions replace Ti^4+^ ions in the crystalline lattice, oxygen vacancies are created to preserve local charge balance. These vacancies contribute to microstrain in the crystallites. Additionally, the formation of O–Fe–O and Fe–O–Ti bonds affects the polarizability and strength of the O–Ti–O bonds, leading to shifts in the peak maxima (Fig. 5).

**Fig. 6:**
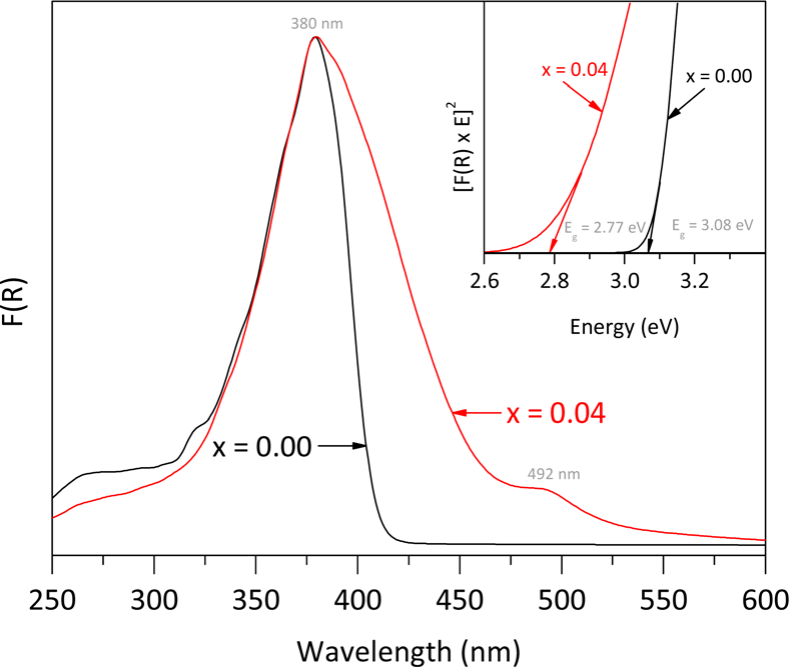
UV-vis DRS spectra of pure rutile and the solid solution with *x* = 0.04 obtained using a PerkinElmer LAMBDA-900 UV/VIS/NIR spectrophotometer equipped with a LABSPHERE® PELA-1020 integrated sphere. The inset represents the curves to get the energy band gap by using Tauc equation.

The UV-vis DRS spectra, shown in Fig. 6, illustrate the effects of Fe doping on the optical properties of the rutile structure. Both the pure rutile and the solid solution with *x* = 0.04 exhibited a strong photo-absorption maximum at a wavelength close to 380 nm. However, the spectrum of the solid solution differs from that of the undoped material, indicating a significant impact of Fe incorporation on the electronic properties. The undoped rutile exhibited UV light absorption with an energy band gap value of approximately 3.08 eV, which corresponds to its intrinsic energy band gap. In contrast, the spectrum of the solid solution revealed a noticeable red shift, likely due to electron transitions between the Fe-3d orbitals and the conduction band of rutile, resulting into a reduction of the energy band gap value from 3.08 to 2.77 eV. Additionally, a second signal was observed in the visible region at approximately 492 nm, associated with the d → d transition, specifically T_2g_ → A_2g_.^29^ Both the red shift and this second signal indicate a notably change in the optical properties of the rutile structure due to Fe incorporation.^30,31^

**Fig. 7:**
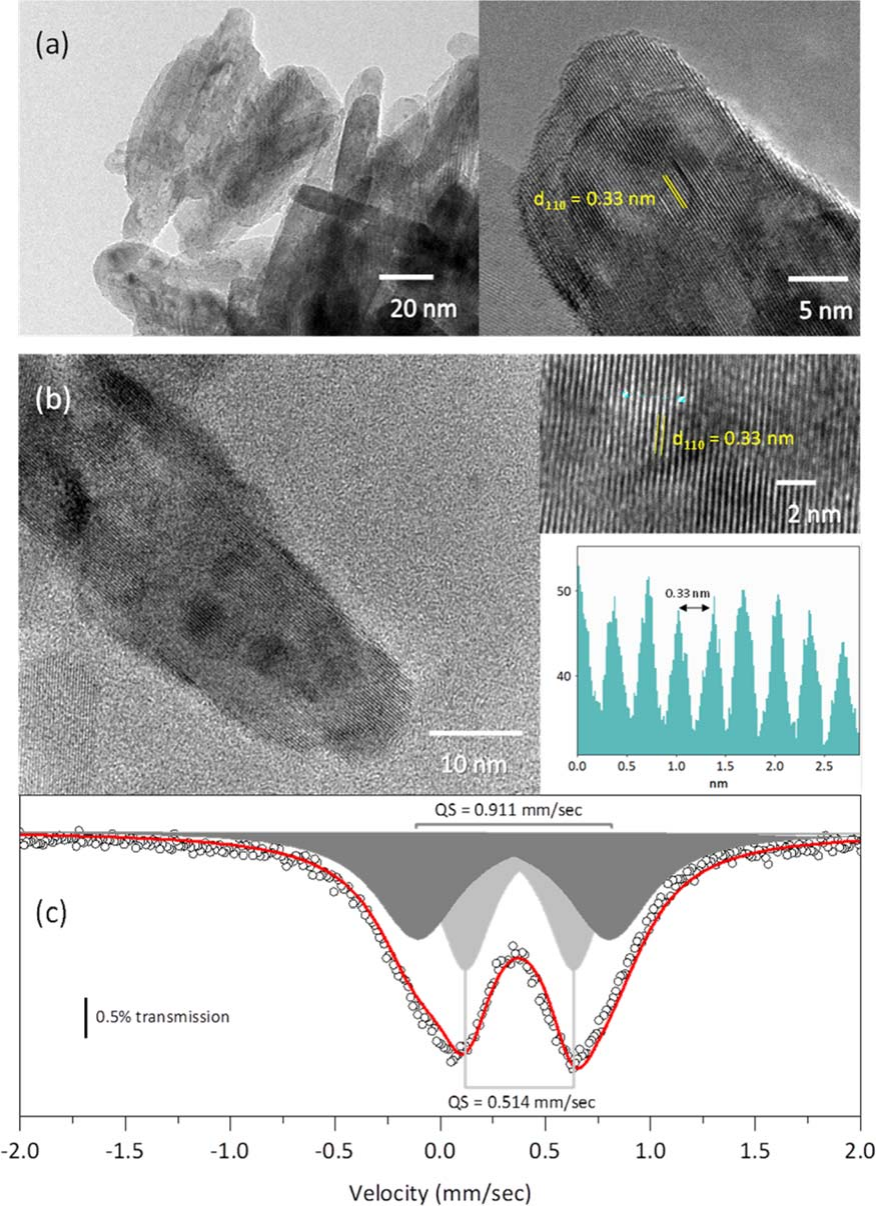
HRTEM images of pure rutile (a) and the solid solution with *x* = 0.04 (b) obtained with a Jeol 2010 electron microscope. Mössbauer spectrum of the solid solution with *x* = 0.04 measured at room temperature (c).

The TEM micrographs of both the rutile and the solid solution with *x* = 0.04 (Fig. 7a and b) exhibit crystallites with a morphology elongated along the *c*-axis, consistent with the morphology observed from the Rietveld refinement. Additionally, the HRTEM micrographs reveal interplanar distances of 0.33 nm, which correspond to the (110) planes of the rutile-like structures.^18^

The Mössbauer spectrum of the solid solution with *x* = 0.04 displayed two doublets: one with an isomer shift of 0.377 mm s^−1^ and a quadrupole splitting of 0.514 mm s^−1^, and another with an isomer shift of 0.349 mm s^−1^ and a quadrupole splitting of 0.911 mm s^−1^. These isomer shifts correspond to Fe^3+^.^32^ The larger quadrupole splitting is likely associated with Fe atoms located on the crystallite surface.

To get further insights into the solid solutions, we constructed a rutile cluster containing 31 titanium atoms, 100 oxygen atoms, and 72 hydrogen atoms. Due to the cluster small size, stabilization required hydroxylation of its surface with OH groups and some water molecules, aligning with the experimental conditions used for synthesizing the solid solutions, which were rich in hydroxyls and water. The cluster's geometry was optimized to its minimal energy using the Density Functional Theory (DFT), with the B3LYP as functional and the 6-31g basis set implemented in the TeraChem code.^33^ The final energy of the cluster was −922,618.146859 eV.

**Fig. 8:**
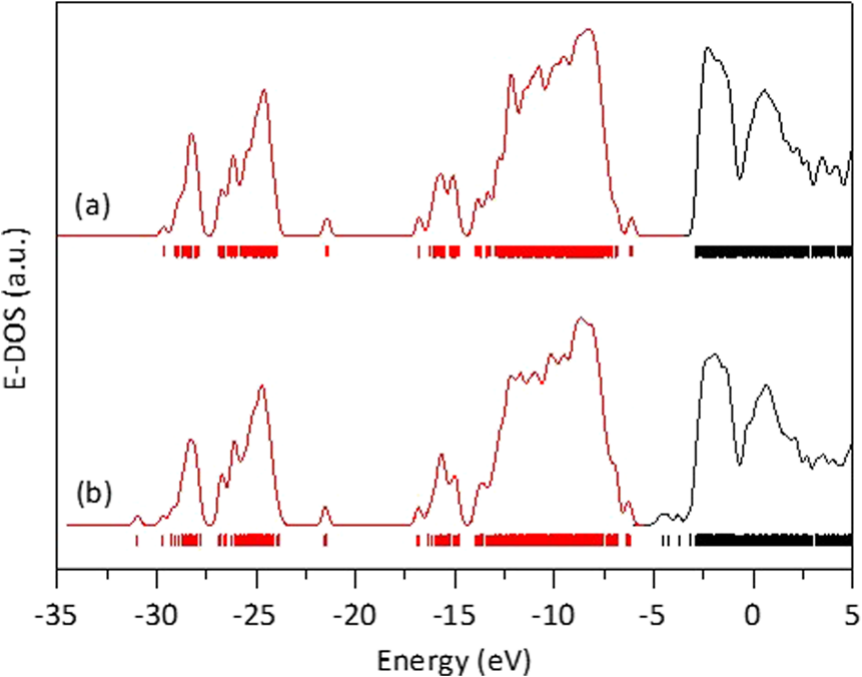
Energy density of states obtained after DFT calculations on a rutile atomic cluster (a), and on an the atomic rutile cluster doped with iron (b).

Starting from this optimized rutile cluster, we constructed a new cluster by substituting three of the Ti atoms with Fe atoms: one on the surface, one on an edge, and one in a corner. Using the DFT method, the geometry of this Fe-doped cluster was optimized to its minimal energy, which was −956,410.227809 eV. This energy is 33 792.080905 eV lower than that of the cluster without Fe, indicating that the Fe-containing cluster is significantly more stable, which aligns with the observed stability of the solid solutions in our experiments. Furthermore, as seen in Fig. 8, the energy band gap for the Fe-free cluster was 3.2327 eV, whereas for the Fe-modified cluster was 1.5973 eV. The reduction in the energy band gap value with Fe doping is attributed to the generation of molecular orbitals having energies below the first non-occupied energy band of pure rutile. The energy of these new orbitals depends on the locations of the Fe-substituted Ti atoms, which in turn affects the calculated energy band gap value.

## 4. Conclusions

In summary, we report the successful synthesis of rutile and the Ti_1−*x*_Fe_*x*_O_2_ solid solutions, with *x* = 0.02, 0.04, 0.06, and 0.08, at 90 °C. Rietveld refinement showed that these solid solutions have a tetragonal crystalline structure and crystallites with elongated morphology along the *c*-axis; the refinement also showed an increase in lattice parameters with Fe content. HRTEM micrographs further corroborated this elongated morphology. Mössbauer spectra identified the presence of two Fe species, both corresponding to Fe^3+^: one located within the bulk another on crystallite surface, providing evidence that Fe atoms are integrated in the solid solution. The experiments demonstrated that the substitution of Ti atoms by Fe atoms in rutile alters its electronic properties. *Ab initio* theoretical calculations demonstrated that this substitution notably enhances stability. Both the experiments and calculations indicated that the presence of Fe reduces the energy band gap, which, according with the calculations, is due the formation of molecular orbitals with energies below the first unoccupied energy band of pure rutile.

## Data availability

The manuscript includes all data supporting this article.

## Author contributions

F. J. Méndez: writing, editing, methodology, investigation. A. Herrera-González: methodology, investigation. A. Morales: methodology, investigation. X. Bokhimi: conceptualization, project administration, funding acquisition, resources, supervision, writing, editing.

## Conflicts of interest

There are no conflicts to declare.
